# Café-Au-Lait Macules in Neurofibromatosis Type 1: Birthmark or Biomarker?

**DOI:** 10.3390/cancers17091490

**Published:** 2025-04-29

**Authors:** Andrea Santangelo, Cristina Chelleri, Marco Tomasino, Mattia Pasquinucci, Francesca Cappozzo, Pasquale Striano, Maria Cristina Diana, Marcello Scala

**Affiliations:** 1 Department of Neurosciences, Rehabilitation, Ophthalmology, Genetics, Maternal and Child Health, University of Genoa, 16132 Genoa, Italy; androsantangelo@gmail.com (A.S.); cristinachelleri@libero.it (C.C.); mattia.pasquinucci@aulss7.veneto.it (M.P.); s5283983@studenti.unige.it (F.C.); strianop@gmail.com (P.S.); 2 Pediatric Neurology and Muscular Diseases Unit, IRCCS Istituto Giannina Gaslini, 16147 Genova, Italy; mcristinadiana@gaslini.org; 3 Cardiology Department, Vall d’Hebron University Hospital, 08035 Barcelona, Spain; marcotomasino21@gmail.com; 4 Department of Medicine, Universitat Autònoma de Barcelona (UAB), 08193 Barcelona, Spain; 5 Medical Genetics Unit, IRCCS Istituto Giannina Gaslini, 16147 Genoa, Italy

**Keywords:** neurofibromatosis, NF1, café-au-lait macules (CALMs), plexiform neurofibromas, early diagnosis, prognostic value

## Abstract

Background: Neurofibromatosis type 1 (NF1) is a rare multisystem disorder characterized by variable expressivity and increased tumor risk. Café-au-lait macules (CALMs) are a hallmark of the disease, often representing one of the earliest clinical manifestations and allowing a clinical NF1 diagnosis if six or more are present. In this study, we aimed to investigate the prognostic value of CALMs at birth in NF1 patients. Methods: We conducted a retrospective study in patients aged ≥ 4 years presenting with CALMs at our Institution between 2020 and 2021, with a minimum follow-up of four years. We retrospectively collected data on CALMs at birth and other clinical manifestations associated with NF1. Results: Among 208 patients evaluated, including 147 with a confirmed diagnosis of NF1, 110 did not show CALMs at birth, and 98 had at least one. The absence of CALMs at birth did not correlate with a lower likelihood of NF1. In contrast, the CALM number at birth directly correlated with the likelihood of NF1, up to 95% in patients with ≥5 macules. Additionally, a higher number of CALMs correlated with a greater prevalence of plexiform neurofibromas (*p* < 0.001). Conclusions: Our findings suggest that a higher number of CALMs may indicate a more severe form of NF1, with an increased risk of plexiform neurofibromas. These results emphasize the importance of a comprehensive evaluation of patients with CALMs, especially in case of multiple lesions, aiming at implementing early NF1 diagnosis, follow-up strategies, and overall patient management.

## Simple Summary

Neurofibromatosis type 1 (NF1) is a rare genetic disorder that affects multiple body systems and increases the risk of tumors. One of its earliest signs is café-au-lait macules (CALMs), pigmented skin spots that can help diagnose NF1 when six or more are present. This study investigated whether CALMs at birth could predict NF1 diagnosis and severity. We analyzed records of children with CALMs, aged four years or older, evaluated at our Institution. Among 208 patients, 147 had confirmed NF1. While some had no CALMs at birth, those with multiple spots were significantly more likely to develop NF1. The risk reached 95% in patients with at least five CALMs at birth. A higher number of CALMs also correlated with an increased risk of plexiform neurofibromas. These findings emphasize the importance of early monitoring for children with multiple CALMs, enabling timely diagnosis, better follow-up, and improved patient care.

## 1. Introduction

Neurofibromatosis type 1 (NF1) is a rare cancer-predisposing syndrome with a wide range of clinical manifestations affecting the skin, nervous system, eyes, and bones [1]. It is an autosomal dominant disorder associated with mutations involving NF1, located on chromosome 17q11.2. Its diagnosis is based on the presence of at least two criteria, including six or more café-au-lait macules (CALMs), axillary or inguinal freckling, two or more neurofibromas or one plexiform neurofibroma (pNF), an optic pathway glioma, Lisch nodules or choroidal abnormalities, distinctive bone lesions, or a pathogenic variant in the *NF1* gene. Additionally, a child with a parent meeting these criteria is diagnosed if they exhibit one or more of these features [2].

NF1 significantly increases the risk of neoplasms. Patients experience a 2.7-fold higher cancer risk and a cumulative malignancy risk of 20% by the age of 50 [3]. Malignant peripheral nerve sheath tumors (MPNSTs) are the most common malignancy, accounting for 3–10% of all soft tissue sarcomas. Among these, 15–70% of cases occur in NF1 patients [4]. The lifetime cumulative risk of developing MPNSTs in NF1 is 8–13% compared with just 0.001% in the general population, making them a leading cause of death in this group [3,5]. Internal plexiform neurofibromas increase the risk of MPNSTs by 20-fold, and 10–50% of MPNSTs arise from malignant transformation of plexiform neurofibromas [6]. Notably, NF1-associated MPNSTs have a more severe prognosis compared to sporadic tumors, with a 5-year survival rate of 16–38% versus 42–57% for non-NF1 cases [3,7,8]. These tumors are highly aggressive and have the highest recurrence rate among sarcomas [9]. Optic pathway gliomas are other common NF1-associated neoplasms, occurring in up to 20% of pediatric cases [10]. These tumors often develop during early childhood and can significantly impact vision and quality of life.

The hallmark feature of NF1 is represented by multiple CALMs. These are flat hyperpigmented skin lesions typically appearing within the first few years of life. Although CALMs can appear in individuals without NF1, the presence of several lesions, especially in combination with other diagnostic features, strongly suggests a diagnosis of NF1 [11]. Given their frequent appearance in early childhood, CALMs are a critical marker for the early diagnosis and management of NF1 [11,12]. The number and size of CALMs can increase over time, especially in the early stages of the disorder. Their early onset represents a pivotal diagnostic tool, as they often appear before more severe NF1-related complications, such as neurofibromas or optic gliomas. While CALMs are a key diagnostic feature, they are not exclusive to NF1. In fact, up to 25% of the general population presents up to three CALMs [13]. Moreover, different disorders can also manifest with multiple CALMs. Legius syndrome is the most important phenocopy of NF1, manifesting with CALMs without the development of neurofibromas or other NF1-related tumors [14]. This phenotypic overlap complicates early diagnosis, particularly in young children. However, molecular testing of the *NF1* gene can differentiate NF1 from other conditions and help guide patient management.

In this study, we aimed at exploring the correlation between the presence of CALMs at birth and the subsequent risk of developing NF1. We also investigated the possible relevance of the presence of CALMs in relation to key prognostic factors of disease outcome, such as the likelihood of neurofibroma development, including plexiform neurofibromas, and other NF1-related complications.

## 2. Materials and Methods

### 2.1. Study Cohort

We retrospectively evaluated pediatric patients presenting with CALMs at birth to assess the risk of developing NF1 in these children. Data were collected from the medical records of subjects followed at the Specialized Center for NF1 Care in IRCCS G. Gaslini, Genoa, between 2020 and 2021. The inclusion criteria for the NF1 group were as follows: age of at least 4 years, the presence of at least one CALM at birth, fulfilling criteria for NF1 diagnosis according to NIH [2], and a minimum follow-up of 4 years at our Institution. Patients with pre-existing genetic diagnoses or conditions that could be confounding factors for a diagnosis of NF1 were excluded from this study. Inclusion criteria for the non-NF1 group included age of at least 4 years, the presence of at least one CALM at birth, clinical and genetic exclusion of NF1, and a follow-up of at least 4 years at our Institution.

### 2.2. Data Collection

Clinical and genetic data were retrospectively collected from electronic medical records. The following information was retrieved: number of CALMs at birth; development of clinical features associated with NF1 during follow-up (including neurofibromas, axillary or inguinal freckling, Lisch nodules, and optic pathway gliomas); family history of NF1 or other genetic disorders, or a genetic confirmation of a variant in *NF1*. Only typical CALMs (homogeneous pigmentation, regular borders) with a minimum diameter of 0.5 cm were considered, in accordance with NIH diagnostic criteria.

All individuals classified as non-NF1 underwent NF1 genetic testing. Conversely, patients with a clinical diagnosis based on ≥2 NIH criteria were included regardless of genetic confirmation.

### 2.3. Statistical Analysis

The CALM number, initially a continuous variable, was categorized into four groups (0, 1–2, 3–4, and 5 or more) based on clinical relevance and data distribution. Summary statistics for categorical variables, stratified by CALM group, were reported as frequencies and percentages. Group comparisons were conducted using the Pearson chi-square test or Fisher’s exact test, as appropriate. Assumptions for the Pearson chi-square test, including the independence of observations and a minimum expected cell count of five in at least 80% of cells, were verified. For cases where these assumptions were not met, Fisher’s exact test was used. Statistical significance was set at two-tailed *p*-values < 0.05. All analyses were performed using Stata software, version 17.0 (StataCorp, College Station, TX, USA).

## 3. Results

### 3.1. Cohort Description

We recruited 208 individuals, including 61 without NF1 and 147 with diagnosed NF1. Patients were categorized by the number of CALMs at birth: 110 with no CALMs, 38 with 1–2 CALMs, 20 with 3–4 CALMs, and 40 with 5 or more CALMs. The Fitzpatrick skin type distribution was recorded for all enrolled subjects. In both NF1 and non-NF1 groups, phototype III was the most frequent, observed in 88 out of 147 NF1 patients (59.9%) and 37 out of 61 non-NF1 patients (60.7%). Phototype II was present in 22 NF1 patients (15.0%) and 10 non-NF1 patients (16.4%), while phototype IV was found in 26 NF1 cases (17.7%) and 9 non-NF1 cases (14.8%). Less frequent were phototype I (four NF1 cases (2.7%), two non-NF1 cases (3.3%)), phototype V (four NF1 (2.7%), one non-NF1 (1.6%)), and phototype VI (three NF1 (2.0%), two non-NF1 (3.3%)). There was no significant difference in the distribution of skin types between the NF1 and non-NF1 groups (*p* > 0.05). The primary characteristics of the cohort are summarized in Table 1.

### 3.2. CALMs’ Correlation with NF1 Diagnosis and pNF

**Figure 1:**
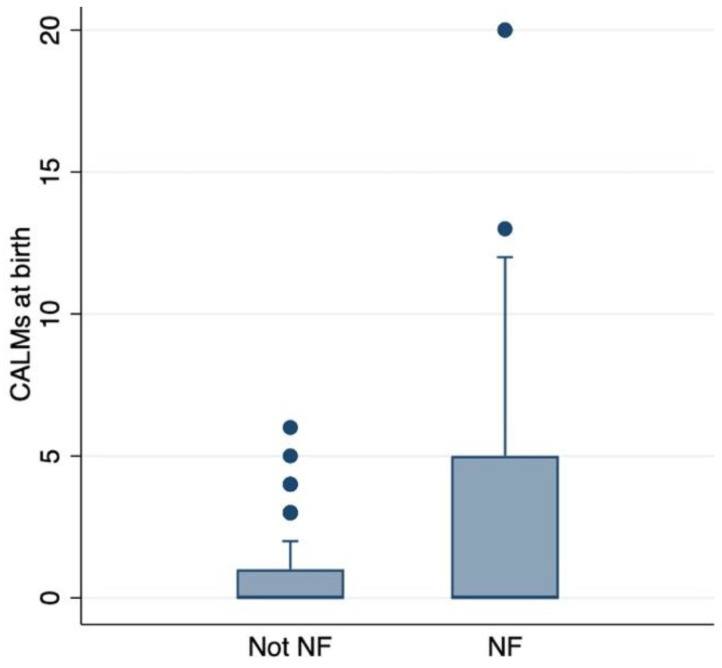
Association between CALMs at birth and NF1 diagnosis. Bar plot graph showing the prevalence of NF1 with an increasing number of CALMs at birth.

The prevalence of NF1 increased with the number of CALMs. A prevalence of 71% (78 patients) was observed in subjects with no CALMs. The prevalence was 50% in subjects with 1–2 CALMs, 60% in those with 3–4 CALMs, and 95% in those with 5 or more CALMs (*p* < 0.001) (Table 1). Interestingly, a significant association was observed between CALM count and the prevalence of pNFs (Figure 1), with a higher prevalence in patients with five or more CALMs (*p* = 0.001) (Table 2).

Additionally, a trend towards higher cutaneous neurofibroma prevalence was noted in the same group (*p* = 0.084) (Table 2).

## 4. Discussion

Beyond their diagnostic value, CALMs may have an important prognostic significance in NF1. In fact, research has already shown that the number and size of CALMs can correlate with disease severity [15], and larger CALMs or a higher number of lesions can occur in patients with more serious complications, such as MPNSTs and skeletal abnormalities [16]. Furthermore, CALMs occurring in patients with NF1 are often more intensely pigmented and larger than those found in individuals with other syndromes or isolated lesions [14,17]. Our findings revealed that children with five or more CALMs exhibited a 95% prevalence of NF1, compared to a 50% prevalence in those with 1–2 CALMs. This aligns with prior studies, such as a retrospective analysis by Ben-Schachar et al., which reported an 80.4% risk of being affected by NF1 in children younger than 29 months with more than six CALMs [18]. Overall, these observations support the relevance of the number and size of CALMs as predictors of disease severity in NF1 patients, highlighting the critical importance of early CALM count assessment as a potential screening tool for NF1.

The early identification of children with a high CALM burden may indeed allow clinicians to make a timely diagnosis, facilitating appropriate intervention in pediatric populations at elevated risk. Early diagnosis can enable tailored surveillance strategies, including genetic testing and regular imaging to monitor for complications such as optic gliomas, neurofibromas, or pNFs. However, not all patients with multiple CALMs will develop a full NF1 phenotype. A review of patients with isolated CALMs revealed that 19.5% to 57.1% of such cases did not meet the clinical criteria for NF1 upon follow-up [11]. This finding highlights the need for molecular confirmation of NF1 in cases where CALMs are the sole presenting feature, especially in the absence of other diagnostic signs.

Notably, none of the patients with negative *NF1* genetic testing developed additional clinical criteria for NF1 during the follow-up period, supporting the reliability of their classification as non-NF1.

Importantly, our findings raise a relevant clinical question regarding children presenting with two CALMs in early infancy, without other signs or genetic confirmation of NF1. While this presentation does not meet diagnostic criteria, we recommend close monitoring due to the potential for additional features to emerge over time. Specifically, we suggest scheduling a clinical re-evaluation at 12 months of age and a dedicated neurological/genetic assessment at 18 months, to ensure the early identification of any evolving NF1 phenotype.

The important impact that an early diagnosis may have on patient management in individuals affected with NF1 has led to the investigation of other possible early markers of disease. For instance, Parrozzani et al. demonstrated the diagnostic value of choroidal abnormalities detected through near-infrared imaging. These defects showed higher specificity than Lisch nodules for early NF1 detection in pediatric patients [19]. Similarly, Cnossen et al. identified minor features such as macrocephaly and thorax abnormalities as predictors of NF1 in children under six. Such markers may be very helpful in trying to enhance diagnostic accuracy when traditional criteria are insufficient [20].

Other early visible cutaneous signs may also support the clinical suspicion of NF1 in infancy. Among these, nevus anemicus and juvenile xanthogranulomas have been reported as additional early markers with diagnostic value in young children [21,22,23]. Notably, these features could be detectable on clinical examination and may aid early stratification, especially when combined with the CALM count.

The increased prevalence of neurofibromas and pNFs in patients with higher CALM counts further highlights the clinical significance of these tumors. Our analysis found that 40% of patients without CALMs exhibited subcutaneous or cutaneous neurofibromas, while this proportion increased to 58% in individuals with 3–4 CALMs. For pNFs, the prevalence increased dramatically from 32% in patients with no CALMs to 63% in those with five or more CALMs. These findings are supported by Nasi et al., who analyzed 63 Greek pediatric NF1 patients and observed that individuals with protein-truncating *NF1* variants presented not only a higher CALM count but also a greater incidence of pNFs [24]. Such genetic predispositions may lead to more severe disease manifestations in NF1 patients with high CALM counts. These observations support the potential utility of CALM assessment in the early clinical stratification and risk management of NF1 patients. The strong association between the number of CALMs and the prevalence of pNFs suggests that CALMs may serve not only as a diagnostic marker, but also as an early indicator of disease severity. As such, CALMs could also be an indicator of the likelihood of developing significant complications. The early identification of individuals with a high CALM burden could therefore enable targeted interventions, such as more frequent imaging studies for the early detection of pNFs or early administration of Selumetinib. The second point is particularly relevant since the side effects of Selumetinib can be age-related [25,26].

NF1 is a multisystem disorder with variable expressivity and elusive genotype–phenotype correlations [1,27,28,29,30]. This also applies to CALMs, where no significant evidence supporting an association between specific genetic variants and their presence or number was reported in the literature. The clinical manifestations of NF1 span diverse systems, including skeletal abnormalities, neurological features, and cardiovascular anomalies. Based on the results of our study, we cannot observe a significant correlation between CALM counts at birth and the presence of these diverse clinical manifestations. Additionally, our findings do not suggest the existence of genotype–phenotype correlations between specific variants in the *NF1* gene and the presence or number of CALMs. This observation highlights the well-known complexity of NF1 and suggests that CALMs alone may not fully capture the multifactorial nature of the disorder. Thus, the multifactorial nature of NF1 prompts the investigation of other potential diagnostic and prognostic markers that could be helpful for implementing clinical diagnosis and management in NF1 patients.

Our study presents some limitations. First, the retrospective design may have limited the availability of information regarding the presence of specific clinical manifestations in the enrolled NF1 patients, such as those that are more strictly age-related. Second, the inclusion in the study cohort of patients enrolled at a single Institution may have generated potential biases in the identification of specific clinical features in NF1 subjects, beyond CALMs, due to the preferential investigation and detection of some disease manifestations based on the expertise of the clinicians. Finally, although previous studies have suggested a possible correlation between CALM size and disease severity, our study did not analyze this parameter systematically. Further prospective investigations are warranted to explore the potential role of macule dimensions in predicting NF1 complications. While every effort has been made to limit the impact of these limitations, these aspects may still affect the generalizability of our findings. Therefore, we recognize the importance of prospective, multicenter studies with larger and more diverse populations to validate our observations and further refine our understanding of the prognostic significance of CALMs.

## 5. Conclusions

In conclusion, our study suggests that CALMs hold prognostic significance in NF1, beyond their diagnostic relevance. This is particularly evident when they are present at birth in higher numbers. Additionally, patients with higher CALM counts showed a significantly increased risk of developing pNFs, which are among the most severe complications associated with NF1. This observation highlights the need for closer surveillance and early intervention for these individuals. Integrating CALM assessment with genetic testing can be a very helpful tool to refine risk stratification in NF1 patients, potentially enabling personalized management and timely treatment. Overall, our findings support the utility of CALMs as a cost-effective tool for guiding early and proactive care strategies towards the improvement of long-term outcomes in NF1 patients.

## Figures and Tables

**Table 1:** Presence of CALMs at birth in NF1 and non-NF1 patients in our cohort.

Number of CALMs	0	1–2	3–4	5 or More
Non-NF1 patients	32 (29%)	19 (50%)	8 (40%)	2 (5%)
NF1 patients	78 (71%)	19 (50%)	12 (60%)	38 (95%)
Total	110	38	20	40

**Table 2:** Comorbidities found in our cohort and their association with CALMs at birth. OPG: optical pathway glioma, pNF: plexiform neurofibroma, ADHD: attention-deficit/hyperactivity disorder, ASD: autism spectrum disorder, CV: cardiovascular.

Comorbidities	CALMs at Birth				
	0 (78 Patients)	1–2 (19 Patients)	3–4 (12 Patients)	5 or More (38 Patients)	*p*-Value
OPG	21 (27%)	4 (21%)	5 (42%)	13 (34%)	0.320
Cutaneous/subcutaneous neurofibromas	31 (40%)	9 (47%)	7 (58%)	21 (55%)	0.084
pNFs	25 (32%)	6 (32%)	6 (50%)	24 (63%)	0.001
Other neoplasms	22 (28%)	3 (16%)	4 (33%)	14 (37%)	0.320
ADHD	9 (12%)	1 (5%)	0	3 (8%)	0.366
ASD	10 (13%)	0	1 (8%)	3 (8%)	0.374
Endocrinological manifestations	30 (38%)	9 (47%)	6 (50%)	20 (53%)	0.134
Allergic manifestations	27 (35%)	10 (53%)	6 (50%)	10 (26%)	0.536
CV manifestations	25 (32%)	5 (26%)	5 (42%)	15 (39%)	0.372

## Data Availability

The data generated by this study are available in the manuscript. Additional information is available from the authors upon reasonable request.
